# Author Correction: On the replicability of diffusion weighted MRI-based brain-behavior models

**DOI:** 10.1038/s42003-025-09178-2

**Published:** 2025-11-21

**Authors:** Raviteja Kotikalapudi, Balint Kincses, Giuseppe Gallitto, Robert Englert, Kevin Hoffschlag, Jialin Li, Christian Büchel, Ulrike Bingel, Tamas Spisak

**Affiliations:** 1 Department of Neurology, University Medicine Essen, Essen, Germany; 2 Center for Translational Neuro- and Behavioral Sciences (C-TNBS), University Medicine Essen, Essen, Germany; 3 Department of Neurology, University Medicine Goettingen, Goettingen, Germany; 4 Department of Diagnostic and Interventional Radiology and Neuroradiology, University Medicine Essen, Essen, Germany; 5 Max Planck School of Cognition, Leipzig, Germany; 6 University Medical Center Hamburg, Hamburg, Germany

Correction to: *Communications Biology* 10.1038/s42003-025-09048-x, published online 30 October 2025

In this article, the author name Kevin Hoffschlag was incorrectly written as Kevin Hofffschlag. The original article has been corrected.

