# Supplementary material for: On the replicability of diffusion weighted MRI-based brain-behavior models
Source: Commun Biol. 2025 Oct 30;8:1512. doi: 10.1038/s42003-025-09048-x (PMC12575672; doi:10.1038/s42003-025-09048-x)
Supplement: Supplementary file 2 — Supplementary information [file 42003_2025_9048_MOESM2_ESM.pdf]

Supplementary material

On the replicability of diffusion weighted MRI-based brain-behavior models

Raviteja Kotikalapudi[1,2,5], Balint Kincses[1,2], Giuseppe Gallitto[1,2], Robert Englert[2,3], Kevin Hofffschlag[1,2], Jialin Li[1,2,4], Christian Büchel[6], Ulrike Bingel[1,2], and Tamas Spisak[2 3*]*

[1] Department of Neurology, University Medicine Essen, Essen, Germany

[2] Center for Translational Neuro- and Behavioral Sciences (C-TNBS), University Medicine Essen, Essen, Germany

[3] Department of Diagnostic and Interventional Radiology and Neuroradiology, University Medicine Essen, Essen, Germany

[4] Max Planck School of Cognition, Leipzig, Germany

[5] Department of Neurology, University Medicine Goettingen, Goettingen, Germany

[6]University Medical Center Hamburg, Hamburg, Germany

**Corresponding author email:* raviteja.kotikalapudi@uk-essen.de, tamas.spisak@uk-essen.de

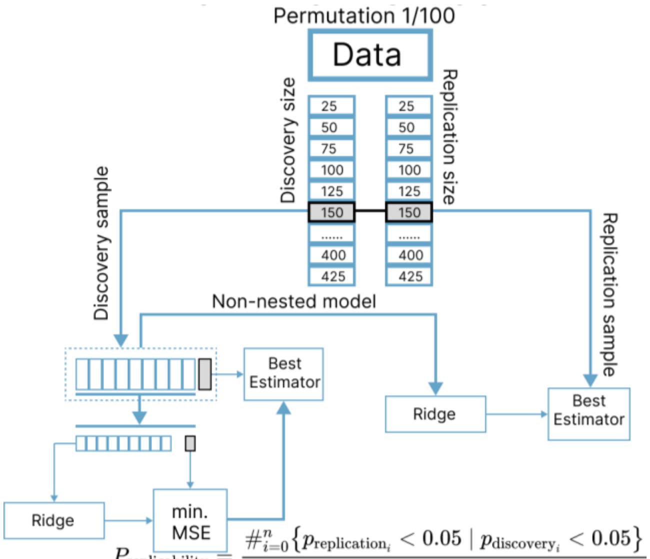

$$P_{\text{replicability}} = \frac{\#_{i=0}^{n}\{p_{\text{replication}_i} < 0.05 \mid p_{\text{discovery}_i} < 0.05\}}{\#_{i=0}^{n}\{p_{\text{discovery}_i} < 0.05\}}$$

Supplementary figure 1. Replicability analysis pipeline. The data split for the discovery and replicability sample is always equal as shown in the figure.

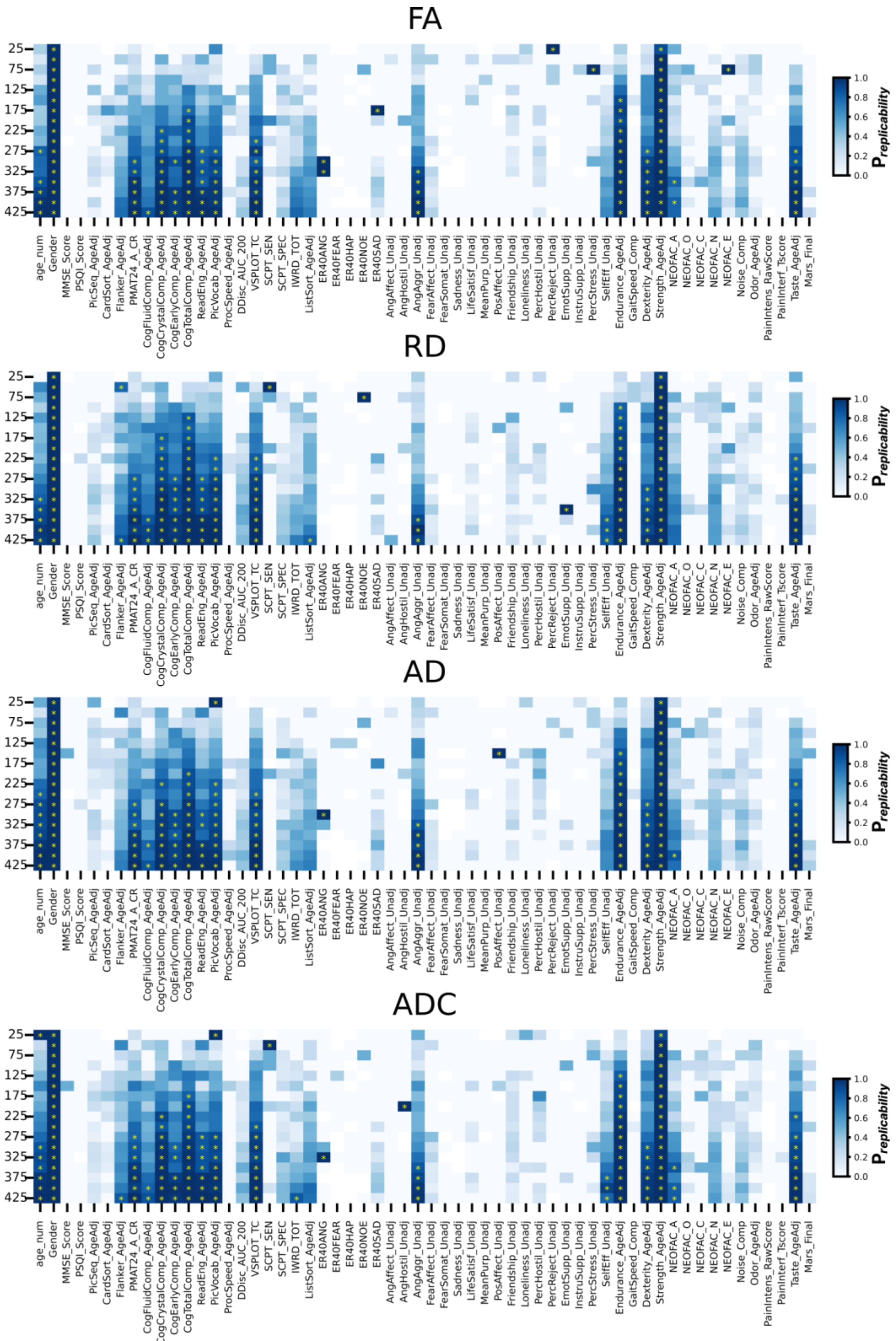

Supplementary figure 2. Sample size requirements for replicable brain–behavior models using DWI in the HCP1200 Dataset.

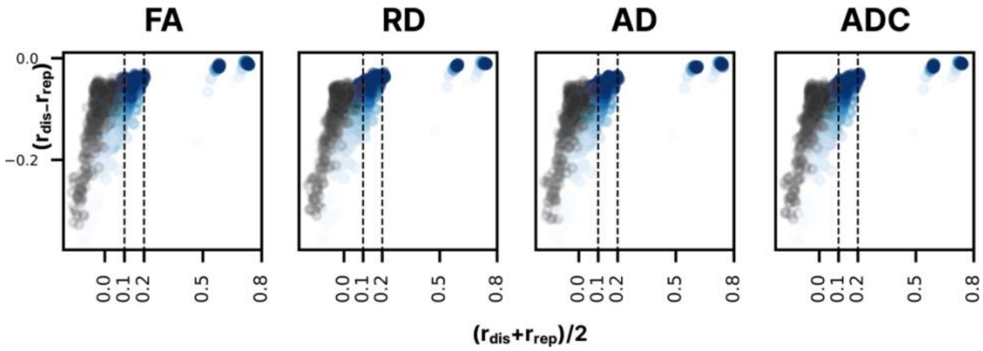

Supplementary figure 3. Effect sizes across sample sizes and phenotypes.

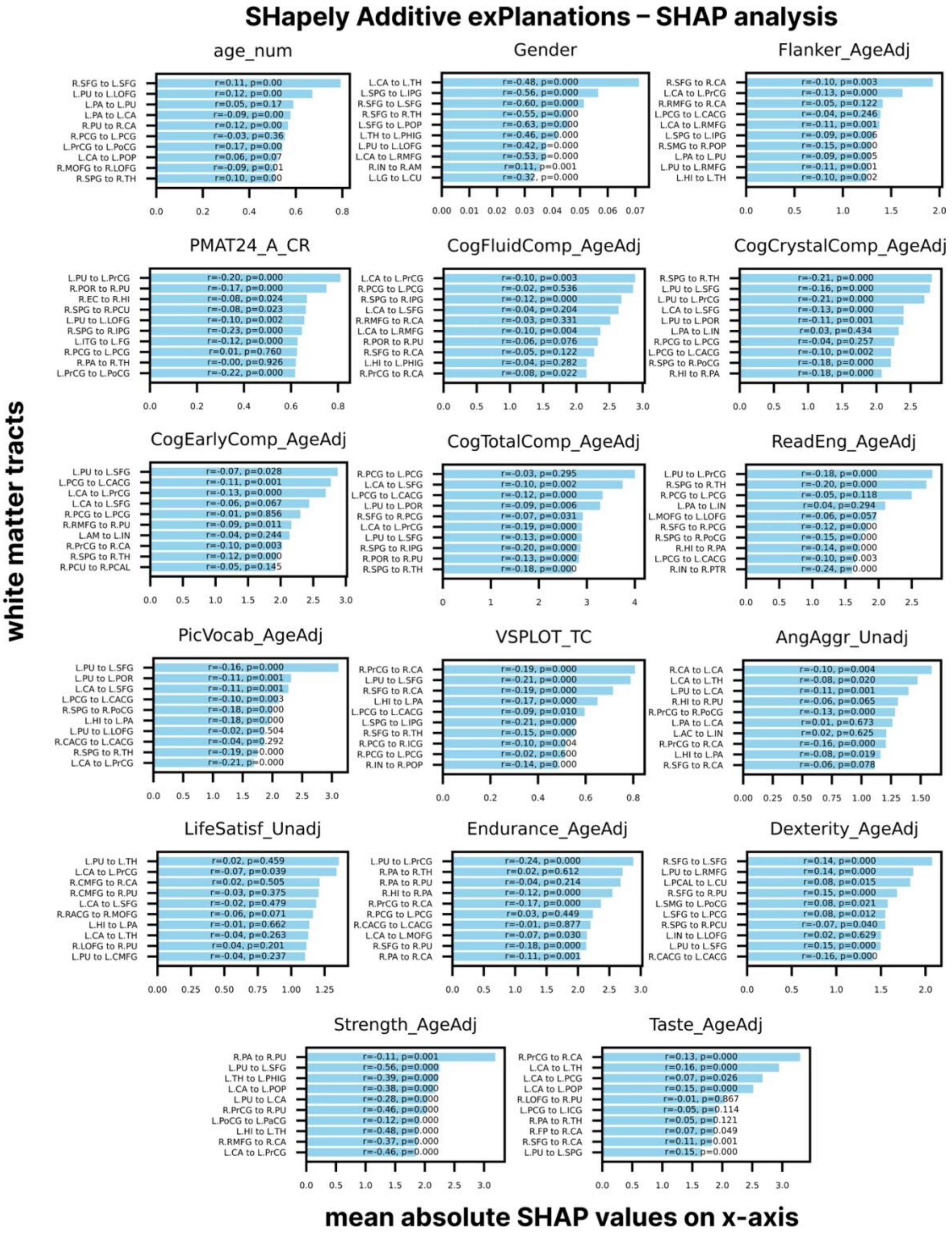

Supplementary figure 4. Interpretation of model weights for insights into mechanisms using the mean absolute SHAP values (bars). Higher values indicate potentially larger drivers of target predictions.

Supplementary table 1. Behavioural phenotypes from the human connectome project (phenotypes=58) and the Amsterdam Open MRI Collection (phenotypes=19).

Human Connectome Project – HCP			
**Domain**	**Sub-domain**	**ID [serial number]**	**Category (State versus trait)**
Alertness	Cognitive Status (Mini Mental Status Exam)	MMSE_Score [1]	State-like
	Sleep (Pittsburgh Sleep Quality Index)	PSQI_Score [2]	
Cognition	Episodic Memory (Picture Sequence Memory)	PicSeq_AgeAdj [3], AgeAdj = age adjusted	Trait-like
	Executive Function/Cognitive Flexibility (Dimensional Change Card Sort)	CardSort_AgeAdj [4]	
	Executive Function/Inhibition (Flanker Inhibitory Control and Attention Task)	Flanker_AgeAdj [5]	
	Fluid Intelligence (Penn Progressive Matrices)	PMAT24_A_CR [6], CogFluidComp_AgeAdj [7], CogCrystalComp_AgeAdj [8], CogEarlyComp_AgeAdj [9], CogTotalComp_AgeAdj [10]	
	Language/Reading Decoding (Oral Reading Recognition)	ReadEng_AgeAdj [11]	
	Language/Vocabulary Comprehension (Picture Vocabulary)	PicVocab_AgeAdj [12]	
	Processing Speed (Pattern Comparison Processing Speed)	ProcSpeed_AgeAdj [13]	
	Self-regulation/Impulsivity (Delay Discounting)	DDisc_AUC_200 [14]	
	Spatial Orientation (Variable Short Penn Line Orientation Test)	VSPLOT_TC [15]	
	Sustained Attention (Short Penn Continuous Performance Test)	SCPT_SEN [16], SCPT_SPEC [17]	
	Memory Verbal Episodic Memory (Penn Word Memory Test)	IWRD_TOT [18]	
	Working Memory (List Sorting)	ListSort_AgeAdj [19]	
Emotion	Emotion Recognition (Penn Emotion Recognition Test)	ER40HAP (happiness)[20], ER40NOE (neutral) [21], ER40SAD (sadness) [22], ER40FEAR (fear) [23], ER40ANG (anger) [24]	State-like
	Negative Affect (Sadness, Fear, Anger)	AngAffect_Unadj [25], AngHostil_Unadj [26], AngAggr_Unadj [27], FearAffect_Unadj [28], FearSomat_Unadj [29], Sadness_Unadj [30]	
	Psychological Well-being (Positive Affect, Life Satisfaction, Meaning and Purpose)	LifeSatisf_Unadj [31], MeanPurp_Unadj [32], PosAffect_Unadj [33]	
	Social Relationships and Social Developments	Friendship_Unadj [34], Loneliness_Unadj [35], PercHostil_Unadj [36], PercReject_Unadj [37], EmotSupp_Unadj [38], InstruSupp_Unadj [39]	
	Stress and Self-Efficacy (Perceived Stress, Self-Efficacy)	PercStress_Unadj [40], SelfEff_Unadj [41]	
Motor	Endurance (2-minute walk test)	Endurance_AgeAdj [42]	Trait-like
	Locomotion (4-meter walk test)	GaitSpeed_Comp [43]	
	Dexterity (9-hole Pegboard)	Dexterity_AgeAdj [44]	
	Strength (Grip Strength Dynamometry)	Strength_AgeAdj [45]	
Personality	Five Factor Model (NEO-FFI)	NEOFAC_A [46], NEOFAC_O [47], NEOFAC_C [48], NEOFAC_N [49], NEOFAC_E [50]	
Sensory	Audition (Words in Noise)	Noise_Comp [51]	
	Olfaction (Odor Identification Test)	Odor_AgeAdj [52]	
	Pain (Pain Intensity and Interference Surveys)	PainIntens_RawScore [53], PainInterf_Tscore [54]	
	Taste (Regional Taste Intensity Test)	Taste_AgeAdj [55]	
	Contrast Sensitivity (Mars Contrast Sensitivity)	Mars_Final [56]	
Demography	Age and Gender	Age [57], Gender [58]	Trait-like
Amsterdam Open MRI Collection – AOMIC			
Demography	Age	age [1]	Trait
	Sex	sex [2]	
	Dominant hand	handedness [3]	
	Body-mass-index	BMI [4]	
	Highest achieved (or) current education level	education_level [5]	
Cognition	IST fluid intelligence subscale	IST_fluid [6]	
	IST memory subscale	IST_memory [7]	
	IST crystallised intelligence subscale	IST_crystallised [8]	
	IST total intelligence scale	IST_total_intelligence [9]	
Personality	BAS drive scale	BAS_drive [10]	
	BAS fun scale	BAS_fun [11]	
	BAS reward scale	BAS_reward [12]	
	BIS scale	BIS [13]	
	Five Factor Model (NEO-FFI)	NEO_N [14] NEO_E [15] NEO_O [16] NEO_A [17] NEO_C [18]	
	Trait anxiety (from the STAI)	STAI_T [19]	

Phenotypes are categorized into two domains: state-like, and trait-like. State-like phenotypes reflect transient fluctuations, such as momentary feelings of sadness or fear. Trait-like phenotypes represent more stable characteristics, like intelligence or physical strength, that change over longer periods compared to state-like phenotypes. Similar categorization was done by Spisak et al in their fMRI brain-behaviour replicability analysis[1].

Supplementary table 2. Performance of non-linear kernel methods of support vector regression and kernel ridge against ridge regression.

	SC replicability (median replicable sample size, mean r)	FA replicability (median replicable sample size, mean r)	RD replicability (median replicable sample size, mean r)	AD replicability (median replicable sample size, mean r)	ADC replicability (median replicable sample size, mean r)	Overall replicability
Ridge	29 (150, .24)	26 (275, .16)	31 (275, .15)	26 (250, .15)	28 (288, .15)	21/58
SVR	28 (150, .22)	24 (275, .15)	28 (275, .15)	24 (275, .15)	24 (275, .15)	18/58
Kernel Ridge	29 (150, .21)	33 (300, .15)	3 (138, .63	14 (163, .19)	9 (250, .19)	20/58

Like the ridge regression, kernel ridge was tuned for the learning rate alpha with values = [1e-4, 1e-3,1 e-2, 0.1, 1e+2, 1e+3, 1e+4] and the kernel was kept at 'rbf' (radial basis function). For SVR, hyperparameter tuning was performed for the regularization strengths with values same as alpha from the other regression models.

Supplementary table 3. HCP – Number of phenotypes replicable with sample size = 425.

Model	Trait-like (# phenotypes = 32)	State-like (# phenotypes = 26)	Total phenotypes = 58
SC | FA| RD| AD | ADC	16	5	21
SC	15	2	17
FA	14	1	15
RD	15	3	18
AD	14	1	15
ADC	13	3	16

SC | FA | RD | AD | ADC = if the phenotype is replicable with at least one of the models.

Supplementary table 4. Replicability across different connectome metrics for the replicable phenotypes in the HCP1200

	**Replicable with SC (yes = 1, no = 0)**	**FA**	**RD**	**AD**	**ADC**	**Total**	**Behavioural category**
**age_num**	1	1	1	1	0	4	Trait
**Gender**	1	1	1	1	1	5	Trait
**Flanker_AgeAdj**	1	0	1	0	1	3	Trait
**PMAT24_A_CR**	1	1	1	1	1	5	Trait
**CogFluidComp_AgeAdj**	1	1	1	1	1	5	Trait
**CogCrystalComp_AgeAdj**	1	1	1	1	1	5	Trait
**CogEarlyComp_AgeAdj**	1	1	1	1	1	5	Trait
**CogTotalComp_AgeAdj**	1	1	1	1	1	5	Trait
**ReadEng_AgeAdj**	1	1	1	1	1	5	Trait
**PicVocab_AgeAdj**	1	1	1	0	0	3	Trait
**VSPLOT_TC**	1	1	1	1	1	5	Trait
**IWRD_TOT**	0	0	0	0	1	1	State
**ListSort_AgeAdj**	0	0	1	0	0	1	Trait
**AngAggr_Unadj**	1	1	1	1	1	5	State
**LifeSatisf_Unadj**	1	0	0	0	0	1	State
**SelfEff_Unadj**	0	0	1	0	1	2	State
**Endurance_AgeAdj**	1	1	1	1	1	5	Trait
**Dexterity_AgeAdj**	1	1	1	1	1	5	Trait
**Strength_AgeAdj**	1	1	1	1	1	5	Trait
**NEOFAC_A**	0	0	0	1	0	1	Trait
**Taste_AgeAdj**	1	1	1	1	1	5	State

Supplementary table 5. Phenotypes replicable across different number of connectome models in HCP1200

	**Replicable across all 5 connectome models (SC, FA, RD, AD, ADC)**	**Replicable across only 1 connectome metric**	**2 metrics**	**3 metrics**	**4 metrics**	**Total**
**Trait-like**	11	2	0	2	1	
**State-like**	2	2	1	0	0	5

Supplementary table 6. HCP – Sample sizes required for significant replication ($p<0.05$).

	SC | FA| RD| AD | ADC, Total phenotypes (=58)					SC | FA| RD| AD | ADC, trait-like (=32)					SC | FA| RD| AD | ADC, state-like (=26)				
Summary	SC	FA	RD	AD	ADC	SC	FA	RD	AD	ADC	SC	FA	RD	AD	ADC
mean	171	243	253	243	262	150	238	225	238	235	325	325	392	325	383
median	150	275	275	250	288	125	275	225	250	250	325	325	375	325	375
min	25	25	25	25	25	25	25	25	25	25	300	325	375	325	350
max	350	425	425	425	425	325	425	425	425	425	350	325	425	325	425

The discovery and the replication splits were always equal.

Supplementary Table 7. Mean sample sizes required for AOMIC datasets

	Sample sizes	$r_{discovery}$	$r_{replication}$
mean	228.12	0.22	0.21
median	225.00	0.19	0.18
min	25.00	0.12	0.12
max	425.00	0.51	0.47

Supplementary table 8. Replicable phenotype sample sizes for FA, RD, AD, ADC-based models in the AOMIC dataset.

**Phenotype**	FA (Sample size, n)	RD	AD	ADC
Sex	100	100	100	100
BMI	325	325	350	325

Supplementary table 9. AOMIC dataset – Comparison of replication using higher resolution atlas with 162 brain regions (Destrieux atlas) versus standard atlas with 82 regions (mrtrix3 labelconvert).

	**Desikan-Killiany (82 regions)**	**Destreux (164 regions)**	**Desikan-Killiany**	**Destreux**
**Phenotype**	Sample size for replication (n)	Sample size for replication (n)	Replicable effect size (Pearson's r)	Replicable effect size (r)
Age	250	250	.167	.167
Sex	25	25	.474	**.530**
BMI	200	200	.188	**.209**
IST_fluid	225	**200**	.164	**.201**
IST_memory	-	**375**	-	**.128**
IST_crystallised	150	**100**	.213	**.219**
IST_intelligence_total	225	**125**	.187	**.214**
BIS	425	**375**	.116	**.130**
NEO_A	325	**300**	.137	**.144**

AOMIC dataset – Comparison of replication results using a higher-resolution atlas (162 brain regions) versus a standard atlas (82 regions, post-MRtrix3 labelconvert). For each phenotype, the sample size required for replication and the replicable effect size (Pearson's *r*) are reported for both the standard and high-resolution atlases. Bold values indicate better performance. Notably, increasing the atlas resolution results in higher sample sizes and effect sizes, suggesting that our results from the HCP1200 cohort, based on the 82-region atlas, may be conservative. Future studies using higher-resolution atlases could potentially observe larger effect sizes, with our model findings serving as a conservative baseline for further exploration.

Supplementary table 10. Streamline connectivity (SC): Partial confounder test for the HCP1200 dataset with TIV as the confounder of interest.

**Phenotype**	**R^2 y and c**	**R^2 y and yhat**	**R^2 yhat and c**	**p-value**	**Ratio of R^2 yhat_c : y_yhat**
age_num	0.03	0.83	0.04	0.128	0.05
Gender	0.39	0.94	0.43	**0.0**	0.46
Flanker_AgeAdj	0.03	0.77	0.05	**0.026**	0.06
PMAT24_A_CR	0.07	0.78	0.12	**0.006**	0.15
CogFluidComp_AgeAdj	0.02	0.75	0.04	**0.014**	0.05
CogCrystalComp_AgeAdj	0.09	0.79	0.14	**0.0**	0.18
CogEarlyComp_AgeAdj	0.04	0.76	0.06	**0.002**	0.08
CogTotalComp_AgeAdj	0.07	0.77	0.11	**0.002**	0.14
ReadEng_AgeAdj	0.07	0.77	0.11	**0.002**	0.14
PicVocab_AgeAdj	0.08	0.8	0.12	**0.0**	0.15
VSPLOT_TC	0.08	0.79	0.13	**0.0**	0.16
IWRD_TOT	0.0	0.74	0.0	0.554	0.0
ListSort_AgeAdj	0.03	0.74	0.05	**0.014**	0.07
AngAggr_Unadj	0.02	0.8	0.03	0.088	0.04
LifeSatisf_Unadj	0.01	0.76	0.02	0.072	0.03
SelfEff_Unadj	0.01	0.77	0.02	0.056	0.03
Endurance_AgeAdj	0.07	0.78	0.11	**0.0**	0.14
Dexterity_AgeAdj	0.01	0.79	0.02	0.136	0.03
Strength_AgeAdj	0.26	0.86	0.34	**0.0**	0.4
NEOFAC_A	0.01	0.79	0.02	0.222	0.03
Taste_AgeAdj	0.03	0.76	0.05	**0.004**	0.07

y = actual observation, yhat = prediction, c = confounder as total intracranial volume. $p<0.05$ indicates a partial confounder effect in the predictive models. Confounder tests were performed using *mlconfound*, a dedicated confounder testing toolbox[2]. **Bold p-value indicates a significant partial confounding effect.**

Supplementary table 11. Streamline connectivity (SC): Full confounder test for the HCP1200 dataset with TIV as the confounder of interest.

**Phenotype**	**R^2 y and c**	**R^2 y and yhat**	**R^2 yhat and c**	**p-value**
age_num	0.03	0.83	0.04	**0.0**
Gender	0.39	0.94	0.43	**0.0**
Flanker_AgeAdj	0.03	0.77	0.05	**0.0**
PMAT24_A_CR	0.07	0.78	0.12	**0.0**
CogFluidComp_AgeAdj	0.02	0.75	0.04	**0.0**
CogCrystalComp_AgeAdj	0.09	0.79	0.14	**0.0**
CogEarlyComp_AgeAdj	0.04	0.76	0.06	**0.0**
CogTotalComp_AgeAdj	0.07	0.77	0.11	**0.0**
ReadEng_AgeAdj	0.07	0.77	0.11	**0.0**
PicVocab_AgeAdj	0.08	0.8	0.12	**0.0**
VSPLOT_TC	0.08	0.79	0.13	**0.0**
IWRD_TOT	0.0	0.74	0.0	**0.0**
ListSort_AgeAdj	0.03	0.74	0.05	**0.0**
AngAggr_Unadj	0.02	0.8	0.03	**0.0**
LifeSatisf_Unadj	0.01	0.76	0.02	**0.0**
SelfEff_Unadj	0.01	0.77	0.02	**0.0**
Endurance_AgeAdj	0.07	0.78	0.11	**0.0**
Dexterity_AgeAdj	0.01	0.79	0.02	**0.0**
Strength_AgeAdj	0.26	0.86	0.34	**0.0**
NEOFAC_A	0.01	0.79	0.02	**0.0**
Taste_AgeAdj	0.03	0.76	0.05	**0.0**

y = actual observation, yhat = prediction, c = confounder as total intracranial volume. $p<0.05$ that the predictions were not fully driven by the confounder. Confounder tests were performed using *mlconfound*, a dedicated confounder testing toolbox[2]. **Bold p-value indicates that the confounder is not fully driving the predictive performance.**

Supplementary table 12. Effects of outliers on replicability analysis.

	SC (mean replicable sample size, mean r)	FA (mean replicable sample size, mean r)	RD (mean replicable sample size, mean r)	AD (mean replicable sample size, mean r)	ADC (mean replicable sample size, mean r)	Overall replicability
Original results	29 (171, .25)	26 (243, .22)	31 (240, .20)	26 (263, .21)	28 (243, .21)	21/58
After outlier correction	31 (189, .25)	28 (250, .21)	29 (253, .21)	26 (263, .22)	28 (235, .21)	19/58

The target measures (phenotypes) were screened for outliers with 3 standard deviations.

Supplementary references

1. Spisak, T., Bingel, U., & Wager, T. D. (2023). Multivariate BWAS can be replicable with moderate sample sizes. *Nature*, *615*(7951), E4-E7.
2. Spisak, T. (2022). Statistical quantification of confounding bias in machine learning models. *GigaScience, 11, giac082.*
