# Supplementary material for: On the replicability of diffusion weighted MRI-based brain-behavior models
Source: Commun Biol. 2025 Oct 30;8:1512. doi: 10.1038/s42003-025-09048-x (PMC12575672; doi:10.1038/s42003-025-09048-x)
Supplement: Supplementary file 3 — Description of Additional Supplementary files [file 42003_2025_9048_MOESM3_ESM.pdf]

Description of Additional Supplementary files

File name: Supplementary Data 1-5

Description: Supplementary data contains the actual effect sizes across all the 5 different connectome measures of SC, FA, RD, AD and ADC.
