# Supplementary material for: On the replicability of diffusion weighted MRI-based brain-behavior models
Source: Commun Biol. 2025 Oct 30;8:1512. doi: 10.1038/s42003-025-09048-x (PMC12575672; doi:10.1038/s42003-025-09048-x)
Supplement: Supplementary file 5 — Reporting summary [file 42003_2025_9048_MOESM5_ESM.pdf]

nature portfolio

Corresponding author(s): Raviteja Kotikalapudi

Last updated by author(s): Aug 5, 2025

Reporting Summary

Nature Portfolio wishes to improve the reproducibility of the work that we publish. This form provides structure for consistency and transparency in reporting. For further information on Nature Portfolio policies, see our Editorial Policies and the Editorial Policy Checklist.

Statistics

For all statistical analyses, confirm that the following items are present in the figure legend, table legend, main text, or Methods section.

n/a	Confirmed	
☐	☒	The exact sample size (*n*) for each experimental group/condition, given as a discrete number and unit of measurement
☐	☒	A statement on whether measurements were taken from distinct samples or whether the same sample was measured repeatedly
☐	☒	The statistical test(s) used AND whether they are one- or two-sided<br>*Only common tests should be described solely by name; describe more complex techniques in the Methods section.*
☐	☒	A description of all covariates tested
☒	☐	A description of any assumptions or corrections, such as tests of normality and adjustment for multiple comparisons
☒	☐	A full description of the statistical parameters including central tendency (e.g. means) or other basic estimates (e.g. regression coefficient) AND variation (e.g. standard deviation) or associated estimates of uncertainty (e.g. confidence intervals)
☐	☒	For null hypothesis testing, the test statistic (e.g. *F*, *t*, *r*) with confidence intervals, effect sizes, degrees of freedom and *P* value noted<br>*Give P values as exact values whenever suitable.*
☒	☐	For Bayesian analysis, information on the choice of priors and Markov chain Monte Carlo settings
☐	☒	For hierarchical and complex designs, identification of the appropriate level for tests and full reporting of outcomes
☐	☒	Estimates of effect sizes (e.g. Cohen's *d*, Pearson's *r*), indicating how they were calculated

Our web collection on statistics for biologists contains articles on many of the points above.

Software and code

Policy information about availability of computer code

Data collection	This study utilized two publicly available datasets: the Human Connectome Project (HCP), downloaded via Amazon Web Services (AWS), and the Amsterdam Open MRI Collection (AOMIC), accessed using DataLad.
Data analysis	The analysis was performed in Python, leveraging libraries such as Scikit-Learn, to implement a multivariate machine learning pipeline. This pipeline was designed to systematically assess the replicability of models that predict behavioral phenotypes using diffusion MRI data. The methodology centered on a rigorous discovery-replication framework, in which models were trained on a "discovery" dataset and then validated on an independent, non-overlapping "replication" dataset. This entire process was iterated across a range of sample sizes to formally calculate the probability of a successful replication, with all analysis scripts shared publicly on GitHub.

For manuscripts utilizing custom algorithms or software that are central to the research but not yet described in published literature, software must be made available to editors and reviewers. We strongly encourage code deposition in a community repository (e.g. GitHub). See the Nature Portfolio guidelines for submitting code & software for further information.

Data

Policy information about availability of data

All manuscripts must include a data availability statement. This statement should provide the following information, where applicable:

- Accession codes, unique identifiers, or web links for publicly available datasets
- A description of any restrictions on data availability
- For clinical datasets or third party data, please ensure that the statement adheres to our policy

The white matter tractography data for actual MRI data will be provided upon reasonable request. The scripts for the statistical analysis for replicability shall be available at https://github.com/pni-lab/dwi-replicability.

Research involving human participants, their data, or biological material

Policy information about studies with human participants or human data. See also policy information about sex, gender (identity/presentation), and sexual orientation and race, ethnicity and racism.

Reporting on sex and gender	Sex was included as a primary demographic phenotype to be predicted from the diffusion-weighted imaging (DWI) data, rather than being treated as a confounding variable. A multivariate model was specifically trained and tested to predict participant gender from their structural connectome, and its replicability was assessed alongside other behavioral and cognitive traits. The analysis revealed that sex was one of the most strongly predictable and highly replicable phenotypes, consistently achieving robust replication with minimal sample sizes investigated across all five DWI metrics.
Reporting on race, ethnicity, or other socially relevant groupings	The primary goal of this research was to assess how reliably structural networks derived from diffusion-weighted imaging can predict human behaviors (e.g., cognitive intelligence) and demographics (e.g., age and sex). While this study confirms the replicability of these predictions, an analysis of dataset diversity was not conducted, and we recommend that future studies incorporate broader socio-demographic factors.
Population characteristics	This study utilized publicly available data from the Human Connectome Project (HCP) and the Amsterdam Open MRI Collection (AOMIC), both of which also focus on characterizing adult populations.
Recruitment	NA
Ethics oversight	NA

Note that full information on the approval of the study protocol must also be provided in the manuscript.

Field-specific reporting

Please select the one below that is the best fit for your research. If you are not sure, read the appropriate sections before making your selection.

☐ Life sciences ☒ Behavioural & social sciences ☐ Ecological, evolutionary & environmental sciences

For a reference copy of the document with all sections, see nature.com/documents/nr-reporting-summary-flat.pdf

Behavioural & social sciences study design

All studies must disclose on these points even when the disclosure is negative.

Study description	This study addresses the challenge of poor replicability in brain-wide association studies by systematically analyzing the sample sizes required to reproduce findings from diffusion-weighted (DWI) MRI data. Using a machine learning pipeline on the public HCP and AOMIC datasets, the researchers employed a rigorous discovery-replication framework to test predictions of various behavioral and demographic traits. The study concludes that while replicability is achievable with moderate sample sizes, success critically depends on the target, with stable, trait-like phenotypes being far more reproducible than transient, state-like ones.
Research sample	Sample size of combining both the datasets, namely HCP and AOMIC =~1700
Sampling strategy	As part of its data splitting strategy, the study randomly shuffled subjects into two equally sized, non-overlapping groups: a "discovery" set for model training and a "replication" set for validation. This random sampling was performed without any explicit demographic matching in order to test a more realistic and stringent form of generalization against arbitrary, unseen subsets of the data. To ensure the robustness of the findings, this entire shuffling and splitting process was repeated 100 times for each phenotype analyzed.
Data collection	Data was collected from two publicly downloadable sources including the human connectome project platform and the OpenNeuro (for the AOMIC study).
Timing	NA

Data exclusions	For Features: The study relied on Ridge regression to manage extreme values in the high-dimensional DWI connectome data (the features). The algorithm's built-in L2 regularization inherently mitigates the influence of potential outliers by shrinking their corresponding model coefficients, thus making the model more robust without the need for manual data removal. For Targets: In a separate, comparative analysis, outliers in the behavioral data (the target variables) were explicitly removed. This was done by excluding any data points that fell more than 3 standard deviations away from the mean for a given phenotype.
Non-participation	Participants missing either diffusion-weighted images or T1-weighted scans were excluded from the analysis.
Randomization	Dedicated confounder analyses were also implemented, for confounders such as total intracranial volume. To facilitate the confounder testing, python package namely, mlconfound was used. mlconfound helps us in understanding the full-confounding and partial confounding of results, given a confounder (c), a target variable (y) and the target estimates (yhat).

Reporting for specific materials, systems and methods

We require information from authors about some types of materials, experimental systems and methods used in many studies. Here, indicate whether each material, system or method listed is relevant to your study. If you are not sure if a list item applies to your research, read the appropriate section before selecting a response.

Materials & experimental systems

n/a	Involved in the study
☒	☐ Antibodies
☒	☐ Eukaryotic cell lines
☒	☐ Palaeontology and archaeology
☒	☐ Animals and other organisms
☒	☐ Clinical data
☒	☐ Dual use research of concern
☒	☐ Plants

Methods

n/a	Involved in the study
☒	☐ ChIP-seq
☒	☐ Flow cytometry
☒	☐ MRI-based neuroimaging

Plants

Seed stocks	NA
Novel plant genotypes	NA
Authentication	NA
